# Semaglutide and High-Intensity Interval Exercise Attenuate Cognitive Impairment in Type 2 Diabetic Mice via BDNF Modulation

**DOI:** 10.3390/brainsci15050480

**Published:** 2025-05-01

**Authors:** Sijie Lai, Zhenghong Kang, Jianting Sun, Ziyu Wang, Yanzi Xu, Sisi Xing, Mengying Feng, Yiyi Wang, Hua Liu

**Affiliations:** 1 College of Sports Medicine, Wuhan Sports University, Wuhan 430079, China; 2 Laboratory of Physical Fitness Monitoring & Chronic Disease Intervention, Wuhan Sports University, Wuhan 430079, China; 3 Hubei Key Laboratory of Exercise Training and Monitoring, Wuhan Sports University, Wuhan 430079, China

**Keywords:** semaglutide, HIIE, diabetes, lactates, cognition

## Abstract

**Background/Objectives**: Diabetes frequently leads to cognitive impairment, encompassing issues with memory and executive function, as well as depression and anxiety. This study examines the impact of high-intensity interval exercise (HIIE) alongside glucagon-like peptide-1 receptor agonist (GLP-1 RA) semaglutide on cognitive dysfunction associated with diabetes. **Methods**: Db/db mice were divided into a control group, semaglutide group, HIIE group, and semaglutide combined with HIIE group to study metabolic and neurobehavioral effects. Cognitive and behavioral tests, hippocampal morphology, and molecular analyses (APP, BDNF, Aβ, p-Tau, PKA, AMPK) were performed. HT22 cells under high glucose were treated with semaglutide, L-lactate, PKA inhibitor H89, and AMPK inhibitor Compound C to validate mechanisms. **Results**: Over 8 weeks, both HIIE and semaglutide improved neuronal morphology and cognitive performance while reducing depression in db/db mice. However, the current study observed no synergistic effects. Both therapies decreased Aβ and p-Tau protein levels and increased BDNF levels in the hippocampus, likely through the AMPK and PKA signaling pathways, respectively. In vitro, HT22 cells under high glucose conditions exhibited elevated APP and p-Tau expression and reduced BDNF levels, which could be altered by L-lactate and semaglutide. The AMPK inhibitor Compound C and the PKA inhibitor H89 attenuated the increase in BDNF levels induced by L-lactate and semaglutide, but their combination mitigated this inhibitory effect. This study suggests that while HIIE and semaglutide improve cognitive function and reduce depression via BDNF, their combined use did not show the anticipated synergistic benefits due to potential antagonism between the AMPK and PKA pathways. **Conclusions**: This has important implications for designing exercise prescriptions for cognitive impairment in diabetics.

## 1. Introduction

Diabetes mellitus (DM) is widely recognized as a significant risk factor for Alzheimer’s disease (AD) due to its effects on vascular dysfunction [1], impaired glucose homeostasis [2], and neuroinflammation [3]. Epidemiological studies have shown that individuals with diabetes have a 1.44 times higher risk of developing mild cognitive impairment (MCI) and a 2.14 times higher risk of developing dementia compared to those without diabetes [4]. Moreover, diabetes has been found to accelerate cognitive decline and shorten the time from MCI to dementia by approximately 3.18 years [4]. A meta-analysis involving 16,584 patients with type 2 diabetes mellitus (T2DM) indicates that intensive glucose control offers more significant cognitive benefits than conventional glucose management [5].

Semaglutide is a long-acting glucagon-like peptide-1 receptor agonist (GLP-1 RA) that has been approved to help people with diabetes and obesity [6]. Unlike other GLP-1 medications, administering semaglutide once a week enhances patient compliance and lessens the burden of treatment [7]. Researchers recently discovered that semaglutide improves insulin signaling in the brain and facilitates synaptic transmission in the hippocampus [8,9]. It is thought that this neuroprotective effect is caused by cyclic adenosine monophosphate (cAMP) signaling, activating protein kinase A (PKA) [10]. Brain-derived neurotrophic factor (BDNF) production is controlled by PKA phosphorylation, which has an impact on neurogenesis and neuroplasticity [11,12,13]. However, cognitive impairment in diabetics tends to develop gradually, and medication alone is often insufficient to halt or reverse its progression [14]. As a result, non-pharmacological interventions are increasingly recognized as essential complements to medication, providing a more effective approach to preventing and treating cognitive decline.

Aerobic exercise [15], resistance training [16], and high-intensity interval exercise (HIIE) [17,18] have been shown to increase serum and hippocampal BDNF levels, effectively reducing depression and cognitive decline. Notably, HIIE elevates lactate levels in the hippocampus, which enhances brain function through the silent information regulator 1 (SIRT1)/PGC1a/FNDC5/BDNF pathway [19]. HIIE also turns on AMP-activated protein kinase (AMPK) in the hippocampus of diabetic rats [20], with hippocampal BDNF contributing to memory restoration via AMPK activation [21]. By improving glucose metabolism and mitigating cognitive decline, exercise is a critical intervention for preventing and treating diabetes. However, several studies suggest that hypoglycemic medications may interfere with the exercise-induced hypoglycemic effects [22,23]. Despite this, the combined impact of exercise and medication on both hypoglycemic levels and diabetes-related cognitive dysfunction, as well as the underlying cellular mechanisms, remains largely unexplored.

This study investigated the synergistic effects of semaglutide and HIIE on enhancing cognitive function in diabetic mice. It specifically analyzed their roles in modulating BDNF expression in the hippocampus via GLP-1 receptor-mediated PKA and exercise-induced AMPK signaling pathways. Furthermore, BDNF expression was assessed in HT22 cells treated with exogenous L-lactate, semaglutide, the AMPK inhibitor Compound C, and the PKA inhibitor H89 under high glucose conditions to illustrate that the lactate-induced AMPK pathway can be inhibited by the PKA pathway.

## 2. Materials and Methods

### 2.1. Experimental Animals

Forty 16-week-old male C57 BL/KsJ db/db mice (Lepr-KO/KO) were purchased from Changzhou Cavens Biogel Company (Changzhou, China). They were randomly assigned to the control group (*n* = 10; CON), the semaglutide group (*n* = 10; SEM), the exercise group (*n* = 10; HIIE), and the combination group (*n* = 10; SEM + HIIE). All animals were kept on a 12:12 dark/light cycle with a humidity of 40% and a temperature of 22 °C. Body weight and blood glucose levels were monitored weekly, while food intake was recorded daily. At the end of the experiment, all animals were anesthetized with isoflurane and euthanized in accordance with institutional animal welfare guidelines. The animal experiments were approved by the Ethics Committee of the Wuhan Sports University of Medical Sciences (Approval No: S0087-20230714-01).

### 2.2. Semaglutide Treatment

The male db/db mice in the SEM and combination groups were administered 0.05 mg/kg of semaglutide subcutaneously every day for 8 weeks [24]. In contrast, the CON and HIIE groups received an equivalent volume of saline solution.

### 2.3. HIIE Intervention

**Figure 1:**
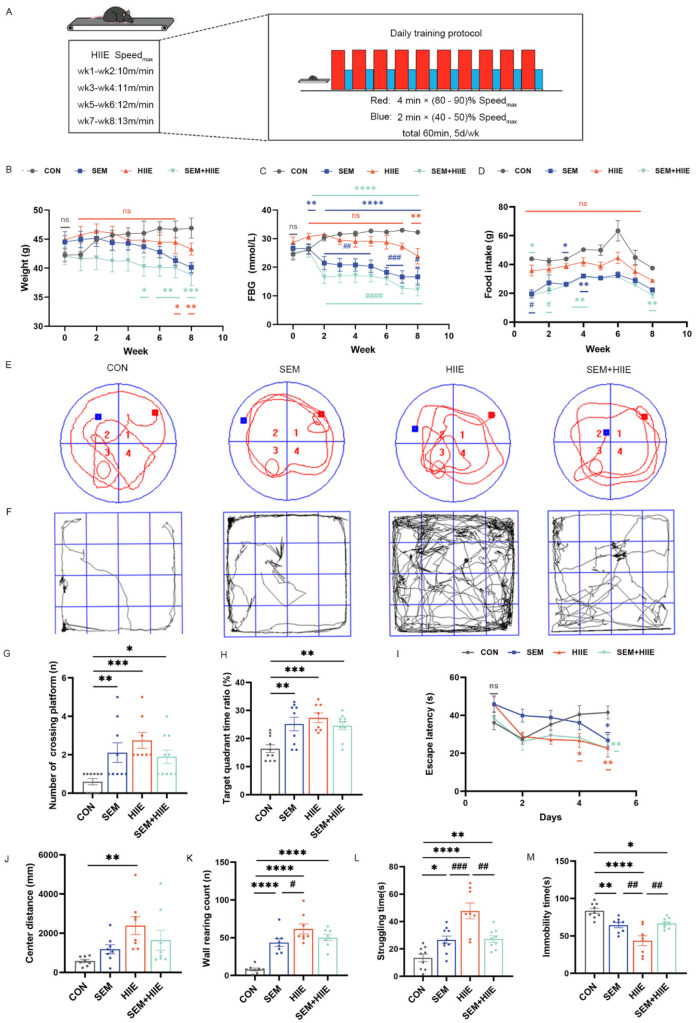
The influence of semaglutide and HIIE interventions on body weight, fasting blood glucose (FBG), food intake, cognitive function, and anxiety-like behavior in db/db mice. (**A**) The protocol of HIIE: 10 cycles of high-intensity exercise, each lasting 4 min (80–90% of maximum speed), interspersed with 2 min active recovery periods at 40–50% of maximum speed; weekly measurements of body weight (**B**) and FBG levels (**C**) in db/db mice; (**D**) average weekly food intake was calculated based on daily food consumption; (**E**) representative swimming track in the Morris Water Maze (MWM) test. Red square represents the starting point, and Blue square represents the goal; (**F**) travel pathway in the Open Field Test (OFT); (**G**) the number of crossings in the target quadrant in the MWM test; (**H**) the ratio of time spent in the target quadrant to total time in the MWM test; (**I**) the escape latency in the MWM test; (**J**) the total distance traveled in the central zone in in the OFT over 10 min; (**K**) the wall-climbing counts in the OFT; (**L**) the struggling time in the Tail Suspension Test (TST); (**M**) the total immobility time in the TST. The data are shown as mean ± SEM; two-way ANOVA, post hoc analysis by Tukey’s multiple comparisons; * *p* < 0.05, ** *p* < 0.01, *** *p* < 0.001, **** *p* < 0.0001 vs. CON, # *p* < 0.05, ## *p* < 0.01, ### *p* < 0.001, #### *p* < 0.001vs. HIIE, and ns: not significant, *n* = 9–11.

The first week of training included a 15 min warm-up at 5 m/min. Based on a previous study [25], the HIIE program consisted of 10 bouts of high-intensity exercise for 4 min (80–90% of maximum speed), with 2 min active rest intervals at 40–50% of maximum speed. Over the 8 weeks, the speed gradually increased from 10 m/min to 13 m/min (Figure 1A).

### 2.4. Behavioral Tests

#### 2.4.1. Open Field Test (OFT)

To track the movement trajectory of mice, an open box measuring 30 cm × 30 cm × 35 cm was utilized, and animal behavior analysis software (Shanghai Gigasoft Technology Co., Ltd., Shanghai, China) was employed for monitoring. The experiment was conducted in a controlled environment that was quiet and dimly lit, ensuring minimal disturbance. Mice were placed at the center of the box, and their activity was observed for a duration of 10 min. During this period, various behavioral metrics were recorded, including the mice’s movement paths, central walking distance, and frequency of climbing. The OFT is typically used to assess exploratory behavior as well as anxiety-like behaviors in rodents, providing insights into their response to novel environments.

#### 2.4.2. Tail Suspension Test (TST)

The mouse’s tail was securely attached to a suspension rod at a point 3/4 of the way from its bottom using medical tape. Once suspended, the mouse’s behavior was monitored using the Topscan behavior analysis system (Clever, San Francisco, CA, USA) to record its struggling and stationary time over a 6 min period. The mouse’s depression status was assessed based on its activity during this observation.

#### 2.4.3. Morris Water Maze (MWM) Test

The MWM tasks were completed in the ninth week following the exercise intervention. The water maze setup included a circular pool (1.6 m in diameter) filled with opaque water, with the water level raised 10 mm above the surface of a hidden platform. Briefly, the mice underwent training sessions, with four trials conducted each day for five consecutive days. On day 6, the platform was removed, and the mice were allowed to swim freely for 60 s. Several parameters were recorded during this probe trial, such as the percentage of time spent in the platform quadrant, the escape latency, and the number of platform crossings. These were all used to test the animal’s spatial memory. Throughout the behavioral testing phase, exercise training continued according to the regular schedule.

### 2.5. Cell Culture and Treatment

HT22 cells were cultured in DMEM high glucose medium (Procell, Wuhan, China) with 1% penicillin (10 kU/mL), streptomycin (10 mg/mL) (Procell, China), and 10% fetal bovine serum (Gibco, Waltham, MA, USA) in a humidified incubator at 37 °C with 5% CO2. HT22 cells were exposed to normal and high glucose media (25 mM and 50 mM), semaglutide (10 nM), PKA inhibitor H89 (10 μM), sodium L-lactate (15 mM), and AMPK inhibitor Compound C (10 μM), and the groups were divided into normal glucose (CON) group, high glucose (HG) group, HG + semaglutide (SEM) group, HG + semaglutide + H89 (SEM + H89), HG + L-lactate (LAC), HG + L-lactate + Compound C (LAC + COM), HG + L-lactate + semaglutide (LAC + SEM), and HG + L-lactate + H89 + semaglutide (LAC + H89 + SEM) groups.

### 2.6. Transmission Electron Microscopy (TEM)

Blood samples were collected from the heart 24 h after the last exercise bout to measure fasting blood sugar (FBS) level. Fresh hippocampal tissue was frozen in liquid nitrogen right away. It was then dehydrated and made clear, and some of it was embedded in paraffin.

The hippocampal tissues were carefully cut into cubes that were about 1 mm × 1 mm × 1 mm. These cubes were then put in 4% paraformaldehyde and 2.5% glutaraldehyde and left on ice for 2 h. The tissues were then washed with PBS and fixed in 1% PBS-buffered osmium tetroxide for 1 h. After dehydration through a graded ethanol series, the tissues were embedded and sectioned into ultra-thin slices. These slices were examined using a transmission electron microscope (H-7650, Hitachi, Ltd., Chiyoda City, Japan).

### 2.7. Hematoxylin–Eosin (HE) Staining

The hippocampal tissues were fixed in 4% paraformaldehyde, then dehydrated, embedded in paraffin, and sectioned. The slices were deparaffinized and stained with HE.

### 2.8. Lactate Levels

The lactate levels in the hippocampus were measured using commercial kits (Solarbio, Beijing, China). The total reaction volume for each reaction was 146 μL, and 10 μL of sample was added.

### 2.9. Quantitative Real-Time Polymerase Chain Reaction (qRT-PCR)

Total RNA from cells and hippocampus tissues was extracted using Trizol reagent (Thermo Fisher, Waltham, MA, USA). Reverse transcription was carried out with the PrimeScript™ RT Reagent Kit (Takara, Dalian, China), and real-time PCR analysis was performed using the QuantiNova SYBR Green PCR Kit (QIAGEN, Hilden, Germany). Primer sequences were as follows: GAPDH, 5′-ATGGGTGTGAACCACGAGA-3′ (forward) and 5′-CAGGGATGATGTTCTGGGCA-3′ (reverse); BDNF, 5′-ATTACCTGGATGCCGCAAAC-3′ (forward) and 5′-CAGTTGGCCTTTGGATACCG-3′ (reverse); and APP, 5′-GCGGCAACAGGAACAACTTT-3′ (forward) and 5′-AACTTTGGGTTGACACGCTG-3′ (reverse). PCR conditions were 95 °C for 2 min, followed by 40 cycles of 95 °C for 5 s and 60 °C for 5 s.

### 2.10. RNA Sequencing

RNA-seq libraries were generated and sequenced by CapitalBio Technology (Beijing, China). The triplicate samples of all assays were constructed into an independent library. The final libraries were quantified using the KAPA Library Quantification kit (KAPA Biosystems, Cape Town, South Africa) and an Agilent 2100 Bioanalyzer (Agilent Technologies, Santa Clara, CA, USA).

### 2.11. Western Blotting (WB)

Hippocampal tissues and HT22 cells were subjected to lysis using radioimmunoprecipitation assay (RIPA) buffer. To separate the proteins, a 10% sodium dodecyl sulfate-polyacrylamide gel (SDS-PAGE) was used. The proteins were then deposited onto a polyvinylidene difluoride (PVDF) membrane. Following blocking with 5% skim milk, membranes were incubated with primary antibodies for BDNF (1:1000, Abcam, Cambridge, UK, Proteintech, Wuhan, China), anti-beta amyloid (1:1000, Abcam), phospho-Tau (1:1000, CST), PKA (1:1000, Proteintech), phospho-PKA (1:1000, Abcam), AMPK (1:1000, CST, Danvers, MA, USA), p-AMPK (1:1000, CST), and GAPDH (1:50,000, Proteintech) at 4 °C overnight. After that, the membranes were treated with the right horseradish peroxidase-conjugated secondary antibodies (1:5000, Jackson ImmunoResearch, West Grove, PA, USA) for one hour at room temperature. ChemiDoc Touch (Bio-Rad, Hercules, CA, USA) was then used to observe the bands.

### 2.12. Statistical Analysis

Data were analyzed using R software (version 3.5.3) and GraphPad Prism (version 7) and are presented as the mean ± SEM. The Shapiro–Wilk test was conducted to assess data normality. For multiple-group comparisons, two-way ANOVA was employed, followed by Tukey’s post hoc test. One-way ANOVA was used to compare average changes among groups, with subsequent Dunnett’s multiple comparisons test. The Student’s *t*-test was applied to determine the differences between the NG and HG groups. Image analysis was performed by ImageJ software (version 1.51w). Statistical significance was considered at *p*-values < 0.05.

## 3. Results

### 3.1. HIIE and Semaglutide Induce Weight Loss and Improve Glycemic Control in db/db Mice

The results for body weight, FBG, and food intake in the db/db mice are shown in Figure 1B–D. Both the SEM and SEM + HIIE groups exhibited significant reductions in body weight compared to the CON group in the last 2–4 weeks (Figure 1B). Specifically, after 8 weeks of intervention, body weight decreased by approximately 10% in the SEM group and 8% in the SEM + HIIE group, whereas the CON group showed an increase of about 11% (Table S1). Additionally, notable decreases in FBG were observed in these groups relative to the CON group in SEM and SEM + HIIE groups from 2 to 8 weeks (Figure 1C), showing a more pronounced reduction compared to the HIIE group (Figure 1C). The food intake in both the SEM and SEM + HIIE groups significantly decreased compared to the control group during the first four weeks (Figure 1D). However, from the fifth week onward, no statistically significant difference was observed until the eighth week; food intake in the SEM + HIIE group started to decrease once again (Figure 1D). The results indicated that both the SEM and SEM + HIIE groups showed the greatest improvements in terms of weight, FBG, and food intake, with no significant difference observed between the two groups.

### 3.2. HIIE and Semaglutide Improve Cognition and Alleviate Depression-like Behavior in db/db Mice

The Morris Water Maze (MWM) test assessed the effects of interventions on spatial learning and memory in mice. The representative swimming track is shown in Figure 1E. The SEM, HIIE, and SEM + HIIE groups had significantly more platform crossings (Figure 1G) and spent more time in the target quadrant (Figure 1H) compared to the CON group. Additionally, these groups showed significantly reduced escape latency on day 5 (Figure 1I).

The Open Field Test (OFT) is commonly used to assess anxiety in mice by measuring exploratory behavior and general activity. In this study, the distance traveled in the central zone showed a significant difference only in the HIIE group compared to the CON group (Figure 1J). Additionally, the number of wall climbs was significantly higher in the intervention groups (Figure 1K), with the HIIE group showing a marked difference compared to the SEM group, indicating that HIIE had a more pronounced effect in alleviating anxiety-like behaviors.

In the Tail Suspension Test, db/db mice in the SEM, HIIE, and SEM + HIIE groups showed significantly increased struggling time (Figure 1L) and reduced immobility time compared to the CON group (Figure 1M). Notably, the HIIE group exhibited the longest struggling time and the shortest immobility time, suggesting that HIIE most effectively alleviated depression-like behavior in db/db mice.

### 3.3. Semaglutide and HIIE Improve Morphology of Hippocampal Neurons in db/db Mice

**Figure 2:**
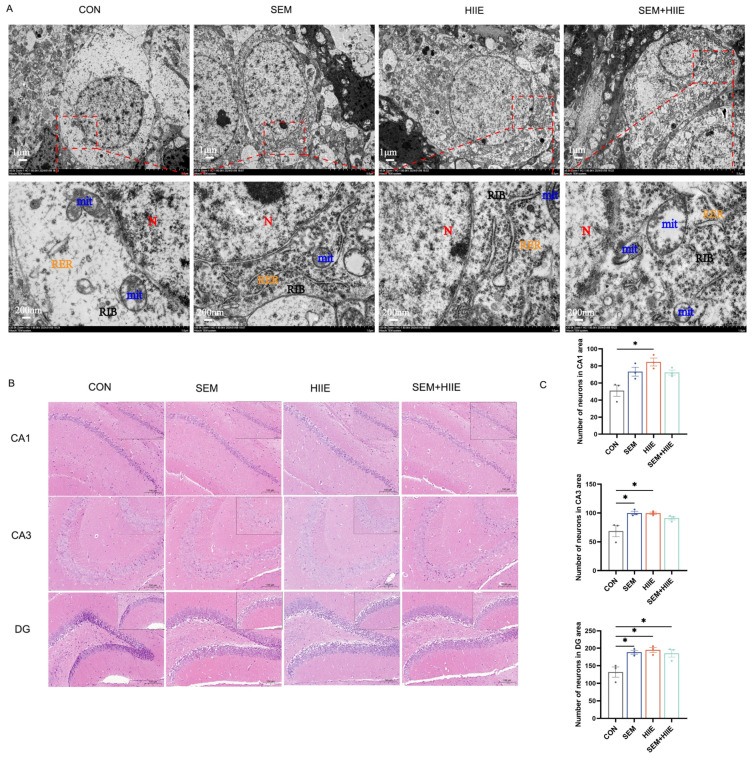
Effects of semaglutide and HIIE interventions on the morphology of hippocampal neurons. (**A**) Transmission electron microscopy images illustrating the ultrastructural damage of hippocampal neurons in db/db mice (Magnifications: 6000× and 30,000×, scale bars: 1 μm and 200 nm). Blue color: mitochondrion (Mit); red color: nucleus (N); yellow color: rough endoplasmic reticulum (RER); black color: free ribosome (RIB). (**B**) Hematoxylin–eosin (HE) staining of hippocampal tissue (magnification: 100× or 200×; scale bars: 100 μm or 50 μm), n = 3. (**C**) The statistical analysis of neuronal counts in the CA1, CA3, and DG regions. The data are shown as mean ± SEM; two-way ANOVA, post hoc analysis by Tukey’s multiple comparisons. * *p* < 0.05, vs. CON, n = 3.

TEM analysis of hippocampal neurons revealed significant ultrastructural changes in the db/db group, including neuronal swelling, decreased ribosome count, and cytoplasmic vacuolation (Figure 2A). In contrast, semaglutide treatment preserved nuclear volume, increased organelle density, and partially restored the endoplasmic reticulum and ribosomes. Neurons in the HIIE group showed numerous intact organelles and no mitochondrial enlargement. The semaglutide + HIIE group displayed mild swelling, slight reduction in nuclear volume, and partial recovery of organelle density. These results suggest that semaglutide and HIIE may help repair hippocampal neuron integrity in db/db mice.

Hematoxylin and eosin (HE) staining was used to observe morphological changes in hippocampal neurons under a light microscope. Both semaglutide and HIIE interventions significantly increased the number of neurons in the CA1, CA3, and DG regions (Figure 2C). These findings suggest that both semaglutide and HIIE effectively mitigate diabetes-induced neuronal loss in these hippocampal regions.

### 3.4. Transcriptome Analysis Reveals Distinct Mechanisms of Semaglutide and HIIE in Improving Diabetic Cognitive Impairment

**Figure 3:**
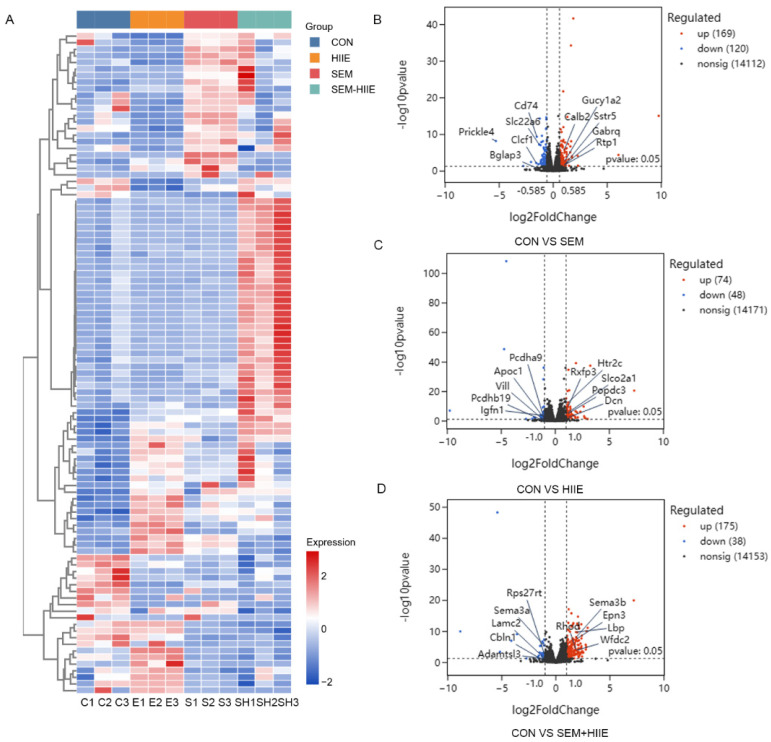
RNA-seq analysis in different groups of db/db mice. (**A**) Multigroup clustering heatmap showing the levels of differentially expressed genes (DEGs) in the hippocampus of db/db mice. Columns represent samples, rows represent genes, and color intensity indicates gene expression levels. C1-3: CON groups; E1-3: HIIE groups; S1-3: SEM groups; SH1-3: SEM + HIIE groups. (**B**–**D**) Volcano plots displaying differentially expressed genes in the hippocampus of the db/db mice in SEM, HIIE, and SEM + HIIE groups. The *x*-axis represents the log2 Fold Change (log2FC) value, while the *y*-axis represents the log10 *p*-value. Red dots represent upregulated genes, and blue dots represent downregulated genes.

**Figure 4:**
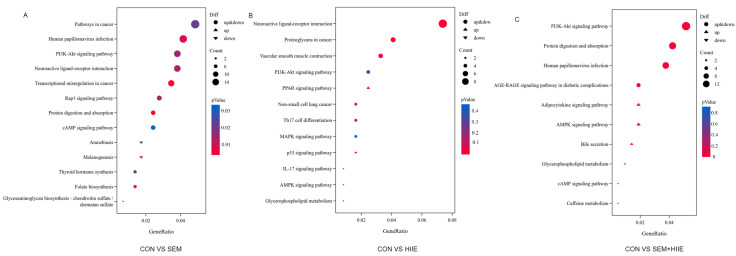
Kyoto Encyclopedia of Genes and Genomes (KEGG) enrichment analysis. (**A**–**C**) Bubble plots showing the top 10 enriched KEGG pathways of DEGs between CON vs. SEM (**A**), CON vs. HIIE (**B**), and CON vs. SEM + HIIE (**C**). The *x*-axis represents the proportion of genes in each entry, and the *y*-axis represents different functional gene categories. Circle size indicates the number of genes enriched in the pathway, and color represents enrichment significance.

Volcano plots and K-means clustering heatmaps (Figure 3A) were used to visualize the distribution of DEGs based on significance and fold change between the SEM, HIIE, and SEM + HIIE groups. Among the top 10 up- and downregulated DEGs, several were involved in environmental information processing, human diseases, metabolism, and organismal systems. Compared to the CON group, differential gene expression analysis revealed 289 DEGs in the SEM group (upregulated: 169, downregulated: 120, Figure 3B), 122 DEGs in the HIIE group (upregulated: 74, downregulated: 48, Figure 3C), and 213 DEGs in the SEM + HIIE group (upregulated: 175, downregulated: 38, Figure 3D), demonstrating distinct transcriptional responses across intervention modalities. The Kyoto Encyclopedia of Genes and Genomes (KEGG) enrichment analysis of DEGs identified key biological pathways, such as those involved in cancer, human papillomavirus infection, PI3K-Akt signaling, neuroactive ligand–receptor interactions, and cAMP signaling, which were among the top 10 significant pathways in the SEM versus CON group (Figure 4A). In the HIIE group, important pathways included how nerves interact with receptors, cancer-related proteoglycans, blood vessel muscle contraction, and PI3K-Akt signaling. The AMPK signaling pathway was also included (see Figure 4B). In db/db mice that were treated with SEM and HIIE, several important pathways were found to be active. These included the PI3K-Akt signaling pathway, protein digestion and absorption, human papillomavirus infection, the AGE-RAGE signaling pathway, the adipocytokine signaling pathway, the AMPK signaling pathway, bile secretion, glycerophospholipid metabolism, and the cAMP signaling pathway related to diabetic complications (see Figure 4C).

### 3.5. HIIE and Semaglutide Increase BDNF and Decrease Aβ, p-Tau Expressions in the Hippocampus of db/db Mice

**Figure 5:**
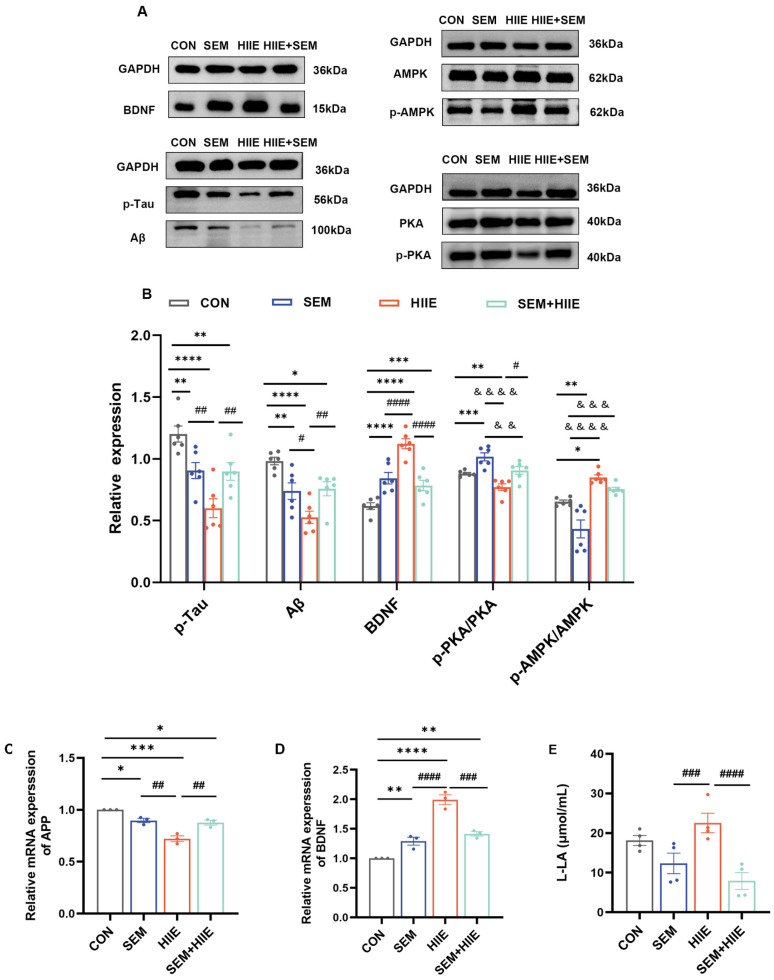
Effects of HIIE and semaglutide on BDNF, Aβ, p-Tau, PKA, and AMPK expression in the hippocampus of the db/db mice. (**A**) Representative Western blot of BDNF, Aβ, p-Tau, PKA, and AMPK expression in hippocampal tissue. (**B**) Quantification graphs of protein expression of BDNF, Aβ, p-Tau, p-PKA/PKA, and p-AMPK/AMPK. *N* = 6. (**C**,**D**) BDNF and Aβ mRNA levels in hippocampal tissue. *N* = 3. (**E**) The level of L-lactate in hippocampal tissue measured using a lactate assay kit. *N* = 4. Data are expressed as mean ± sem. Statistical analysis was conducted using two-way ANOVA. * *p* < 0.05, ** *p* < 0.01, *** *p* < 0.001, **** *p* < 0.0001 vs. CON; & *p* < 0.05, && *p* < 0.01, &&& *p* < 0.001, &&&& *p* < 0.0001 vs. SEM; # *p* < 0.05, ## *p* < 0.01, ### *p* < 0.01, #### *p* < 0.01 vs. HIIE.

To evaluate the effects of HIIE and semaglutide on diabetes-induced cognitive impairment, the current study measured the expression of Aβ, p-Tau, BDNF, PKA, and AMPK proteins in the hippocampus of db/db mice (Figure 5A). The SEM, HIIE, and SEM + HIIE groups had higher levels of BDNF and lower levels of Aβ and p-Tau (Figure 5B), and similar results were seen at the mRNA level (Figure 5C). Notably, the HIIE group exhibited the highest BDNF mRNA and protein expression (Figure 5B,D) and the lowest Aβ and p-Tau levels among all intervention groups (Figure 5B).

### 3.6. HIIE and Semaglutide Regulate L-Lactate, p-PKA/PKA, and p-AMPK/AMPK Expressions in the Hippocampus of db/db Mice

The HIIE group demonstrated the lowest expression of p-PKA/PKA (Figure 5B) and the highest expression of p-AMPK/AMPK (Figure 5B) among all groups. Additionally, hippocampal L-lactate levels were significantly elevated in the HIIE group compared to the SEM and SEM + HIIE groups (Figure 5E).

### 3.7. Lactate and Semaglutide Regulate PKA, BDNF, and AMPK Expression in HT22 Cells

**Figure 6:**
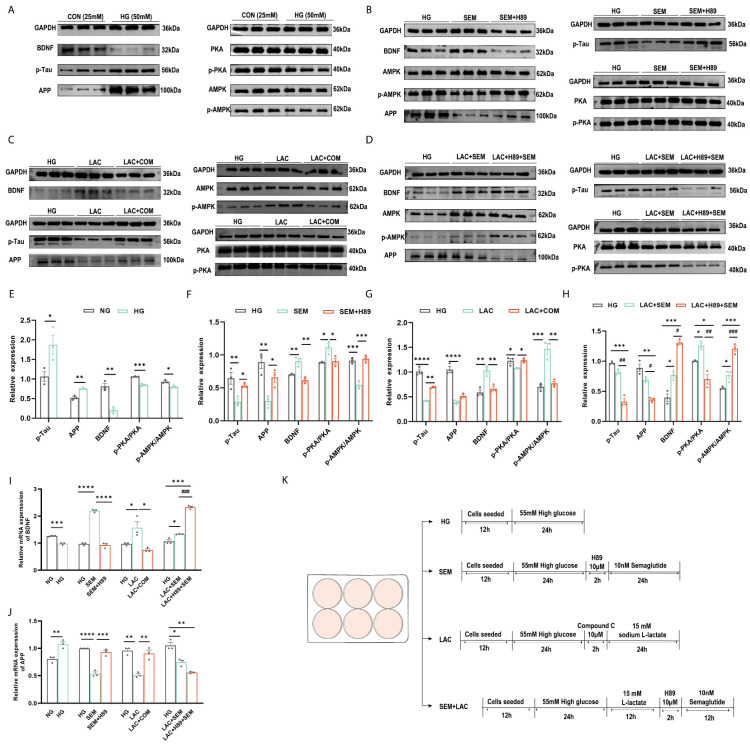
Lactate and semaglutide modulated the PKA, BDNF, and AMPK expressions in HT22 cells. (**A**–**D**) Representative Western blot of p-Tau, APP, BDNF, AMPK, p-AMPK, PKA, and p-PKA in HT22 cells. (**A**) HT22 cells treated with HG media; (**B**) HT22 cells treated with semaglutide and the PKA inhibitor H89; (**C**) HT22 cells treated with L-lactate and the AMPK inhibitor Compound C; (**D**) HT22 cells treated with L-lactate, semaglutide, and H89; (**E**–**H**) quantification graphs of protein expression of BDNF, APP, p-Tau, p-PKA/PKA, and p-AMPK/AMPK in HT22 cells treated by semaglutide and H89 treatment; (**G**) quantification graphs of the protein expression flowing L-lactate and Compound C treatment; (**H**) quantification graphs of the protein expression flowing L-lactate, semaglutide, and H89; (**I**,**J**) mRNA levels of BDNF and APP in HT22; (**K**) cell experiment flowchart. Data are expressed as mean ± sem (*n* = 3). * *p* < 0.05, ** *p* < 0.01, *** *p* < 0.001, **** *p* < 0.0001. # *p* < 0.05, ## *p* < 0.01, ### *p* < 0.01, #### *p* < 0.01.

To explore the mechanisms by which lactate and semaglutide alleviate diabetes-induced cognitive impairment, this study treated HT22 cells under high-glucose (HG) conditions with the AMPK inhibitor Compound C and the PKA inhibitor H89. HG exposure significantly increased p-Tau and APP levels while reducing BDNF, p-AMPK/AMPK, and p-PKA/PKA expression compared to normal glucose (NG) conditions (Figure 6A,E).

Under high glucose conditions, semaglutide treatment significantly increased BDNF levels and the p-PKA/PKA ratio while reducing p-AMPK/AMPK, p-Tau, and APP levels (Figure 6B,F). The PKA inhibitor H89 confirmed that PKA promoted BDNF production. In contrast, lactate treatment elevated BDNF and p-AMPK/AMPK levels but decreased p-PKA/PKA, p-Tau, and APP levels (Figure 6C,G). The AMPK inhibitor Compound C confirmed that AMPK enhanced BDNF expression.

To further examine the neuroprotective effects of high-intensity interval exercise (HIIE) combined with semaglutide, the current study co-treated HT22 cells with lactate, semaglutide, and H89 under HG conditions. This combination greatly raised the levels of BDNF and p-AMPK/AMPK, while lowering the levels of p-PKA, APP, and p-Tau (Figure 6D,H). These findings suggest that PKA inhibition by H89 attenuates semaglutide’s effect on AMPK, preserving lactate’s cognitive benefits. 

## 4. Discussion

In this study, the interactive effects of semaglutide and HIIE interventions on the improvement of cognition in diabetic mice were observed. Following eight weeks of exercise and medication treatments, the db/db mice effectively induced weight loss and improved glycemic control, respectively. Behavioral tests indicated that both semaglutide and HIIE enhanced memory and alleviated depression-like behavior. Surprisingly, the combined treatment did not boost cognitive improvements as expected. Instead, it reduced the benefits related to memory and depression in the OFT and Tail Suspension Tests. Mechanistically, hippocampal RNA sequencing suggested that semaglutide and HIIE exerted their effects through the PKA and AMPK signaling pathways, potentially causing antagonism in regulating BDNF, as confirmed by in vitro experiments.

Effective diabetes management relies on three primary strategies: dietary restrictions, routine physical activity, and pharmacologic therapy, all of which contribute to glycemic control and cardiovascular risk reduction [26,27]. Studies have demonstrated that chronic aerobic exercise improves cognitive function by regulating plasma BDNF levels, enhancing glucose tolerance, and reducing inflammation [28,29,30]. However, studies comparing high-intensity interval exercise (HIIE) and moderate-intensity continuous training found no significant differences in cognitive function or BDNF levels in middle-aged overweight men [31]. Notably, an 8-week HIIE intervention ameliorated Alzheimer’s disease (AD) pathology in the hippocampus of T2DM rats [20]. Beyond exercise, pharmacologic interventions such as semaglutide have demonstrated promising neuroprotective effects. Studies show that using semaglutide for patients with type 2 diabetes and obesity can lower the risk of being diagnosed with AD for the first time and may help delay its starting point [32]. Moreover, emerging evidence suggests that semaglutide mitigates neuroinflammation [33], increases neurogenesis [34], and provides broader neuroprotective benefits [35], making it a potential therapeutic option for cognitive dysfunction. Notably, sex differences may influence both cognitive function and metabolic regulation. Female db/db mice are more prone to hyperglycemia than males [36], and a rapid decline in estrogen activity may contribute to the interplay between diabetes and dementia [37]. Estrogen also promotes BDNF expression via the classical ERα-mediated transcriptional pathway [38], potentially impacting diabetes-related neurocognitive outcomes. While both exercise and semaglutide have been independently studied for cognitive enhancement, limited research has explored their combined effects. Investigating their interaction could provide valuable insights into optimizing therapeutic strategies for cognitive decline in diabetic populations.

The current study found that HIIE, semaglutide, and their combined intervention independently influenced cognitive function and behavior in the MWM, OFT, and TST, aligning with changes in p-Tau, Aβ, and BDNF protein levels in the hippocampus of db/db mice. BDNF levels in serum, cerebrospinal fluid, and brain tissue are widely used as biomarkers for cognitive impairment and depressive disorders [13,39,40,41]. Notably, eight weeks of HIIE induced the most significant improvements in anxiety-like behavior compared to the combined intervention. Individuals with diabetes often experience anxiety and depression, significantly affecting their quality of life. Engaging in regular exercise has been found to have a positive impact [42,43], with acute elevations in BDNF contributing to neuroprotective and neurotrophic effects [44]. Additionally, exercise combined with standard treatment has been reported to improve glycemic control and reduce depression and anxiety in elderly T2DM patients by enhancing serotonin (5-HT) and norepinephrine (NE) expression [45]. TST data showed that HIIE was more effective than the combined intervention in depression. This might be because of the side effects of semaglutide. While semaglutide regulates appetite, enhances satiety, promotes insulin secretion, and supports weight loss [46], it has also been linked to adverse mood changes [47]. Exercise suppresses appetite through modulators such as lactate, BDNF, sex hormones, growth differentiation factor 15 (GDF15), and asprosin [48], but is not closely related to GLP-1 in individuals with obesity [49]. Therefore, the combination of exercise with semaglutide may have a synergistic effect on negative emotions, reducing exercise-induced effectiveness in alleviating depressive behavior.

Furthermore, RNA-seq data revealed distinct pathway activations in different treatment groups. The semaglutide group had the cAMP signaling pathway as one of the top 10 different pathways, but the AMPK pathway was not present. In contrast, the exercise group activated the AMPK pathway but lacked cAMP signaling. Notably, the combined group displayed both pathways. GLP-1, which targets the cAMP/PKA pathway, is considered a promising therapeutic strategy for type 2 diabetes mellitus (T2DM) and neural modulation [50,51]. Previous studies have shown that GLP-1 activates the adenylate cyclase (AC)/cAMP/PKA pathway, enhancing calcium channel activity [52] and promoting cAMP-response element binding protein (CREB) phosphorylation, thereby exerting neuroprotective effects [53]. Meanwhile, HIIE mitigates diabetes-related cognitive decline via the AMPK pathway, alleviating hippocampal impairments [20,54]. As a conserved serine/threonine kinase, AMPK regulates glucose and lipid metabolism, promotes hippocampal BDNF induction, and plays a key role in managing diabetes and its complications [21,55]. The relationship between PKA and AMPK signaling is complicated, as they can work against each other or together, depending on the type of cell. For instance, luteinizing hormone (LH)/PKA reduces AMPK phosphorylation at Thr172, whereas AMPK can inhibit LH/PKA-induced progesterone production in luteal cells [56]. Additionally, AMPK-mediated phosphorylation of β-arrestin-1 at Ser330 enhances phosphodiesterase 4 activity, attenuating β-adrenergic receptor signaling and cAMP/PKA activation, which may benefit cardiac function [57]. In adipocytes, PKA phosphorylation of AMPKα1 at Ser173 suppresses AMPK activity, thereby modulating lipolysis [58]. However, under oxidative stress induced by diabetes and ischemia, AMPK and dual specificity A-kinase-anchored protein 1 (D-AKAP1)/PKA collaborate to regulate mitochondrial function in endothelial cells, cardiac cells, and neurons [59]. In the diabetic hippocampus, the specific PKA phosphorylation site affecting AMPK remains unclear. Our findings suggest a potential antagonistic relationship between exercise and medication in cognitive improvement and depression alleviation, highlighting the need for further investigation.

Enhanced global PKA activity can counteract the protective effects of AMPK under chronic stress and degenerative conditions. PKA and AMPK exhibit a bidirectional regulatory relationship in mitochondrial dynamics: PKA, targeted to the outer mitochondrial membrane via D-AKAP1, promotes mitochondrial fusion and neuroprotection, whereas AMPK phosphorylates D-AKAP1 under oxidative stress, leading to its dissociation from PKA and promoting mitochondrial fission and mitophagy [59]. Cytosolic PKA has been shown to antagonize AMPK signaling in insulin-responsive cells and attenuate the glucose-lowering effects of metformin [60,61]. Mechanistically, this inhibition is mediated by increased inhibitory phosphorylation of AMPK at Ser173 and reduced activating phosphorylation at Thr172 [61]. Although specific PKA phosphorylation sites on AMPK in the diabetic hippocampus remain unclear, PKA has been reported to suppress AMPK activity through phosphorylation at Ser173, Ser485/491, or Ser497 [62]. Additionally, lactate inhibits adenylyl cyclase activity via activation of G protein-coupled receptor 81 (GPR81), thereby reducing cAMP levels and downstream PKA signaling, including the PKA–CREB axis [63,64]. GLP-1 receptor activation relies not only on PKA signaling to enhance insulin secretion but also inhibits the AMPK pathway to improve insulin sensitivity [65]. These findings suggest that PKA-mediated inhibition of AMPK may contribute to the differential outcomes observed in diabetic hippocampal tissue, potentially explaining why the application of HIIE without semaglutide treatment might appear to be more effective.

This study explored the interaction between lactate, AMPK, and PKA in HT22 cells under high glucose conditions using Compound C and H89 to precisely regulate cellular responses. Metformin-induced AMPK activation has been shown to enhance the BDNF/P70S6K pathway in hippocampal neurons, improving learning and memory [12]. Similarly, high-intensity exercise elevates lactate levels, which correlate with increased hippocampal and peripheral BDNF expression [66,67]. Lactate, an exercise-induced myokine, penetrates the blood–brain barrier (BBB) and triggers the BDNF pathway, potentially safeguarding against cognitive decline and neurodegeneration through the SIRT1/PGC1/FNDC5 pathway [19,68,69]. Exercise also upregulates brain AMPK, further promoting BDNF through the AMPK/SIRT1 pathway [70]. While lactate is known to activate AMPK in skeletal muscle [71], kidneys [72], and liver [73], its effects on neural cells remain underexplored. Our findings demonstrate that lactate activates AMPK in hippocampal neurons, increasing BDNF, APP, and p-Tau levels—a process blocked by Compound C. However, when lactate and semaglutide were co-cultured in HT22 cells under high glucose conditions, BDNF levels increased while APP and p-Tau decreased. Notably, H89 enhanced these effects, indicating that inhibition of PKA increased the binding of high-intensity exercise and semaglutide to BDNF expression.

A limitation of this study is its reliance on protein inhibitors in vitro and lack of direct in vivo validation in db/db mice. While we confirmed that semaglutide and HIIE improve cognitive function in diabetes through distinct mechanisms, their key interaction remains unclear. Based on PPI results, we need more animal studies to better understand how the PKA and AMPK pathways affect cognitive decline in diabetes, considering potential sex differences in metabolic and neuroendocrine regulation. Moreover, interspecies differences in metabolism, drug response, and exercise adaptation, along with our limited sample size, may affect the generalizability of the findings. Future research, including clinical trials, is essential to enhance translational relevance.

## 5. Conclusions

Our findings demonstrate that both HIIE and semaglutide mitigate diabetes-induced neuronal damage, improving learning and memory in db/db mice via the AMPK/BDNF and PKA/BDNF pathways, respectively. However, their combined intervention offered no significant cognitive or antidepressant advantage over single treatments. Notably, the exercise-only group showed the greatest improvement in depression. These insights deepen our understanding of how HIIE and semaglutide influence diabetes-related cognitive decline and depression. When prescribing exercise for diabetic individuals, the interaction between pharmacological treatments and exercise modalities must be carefully considered.

## Data Availability

The datasets used and analyzed during the current study are available from the corresponding author on reasonable request.

## Supplementary Materials

The following supporting information can be downloaded at: https://www.mdpi.com/article/10.3390/brainsci15050480/s1, Table S1: Changes in body weight and blood glucose levels before and after intervention.
