# Supplementary material for: Semaglutide and High-Intensity Interval Exercise Attenuate Cognitive Impairment in Type 2 Diabetic Mice via BDNF Modulation
Source: Brain Sci. 2025 May 1;15(5):480. doi: 10.3390/brainsci15050480 (PMC12109977; doi:10.3390/brainsci15050480)
Supplement: Supplementary file 1 [file brainsci-15-00480-s001.zip › brainsci-3566528-supplementary.pdf]

Table S1. Changes in body weight and blood glucose levels before and after intervention.

Group	Baseline Body Weight (g, Mean±SEM)	Post-Intervention Body Weight (g, Mean±SEM)	Body Weight Change (%)	Baseline Blood Glucose (mmol/L, Mean±SEM)	Post-Intervention Blood Glucose (mmol/L, Mean±SEM)	Blood Glucose Change (%)
CON	42.24 ± 1.12	46.91 ± 1.74*	11%	24.56 ± 1.99	32.24 ± 0.76**	31%
SEM	44.51 ± 1.78	40.14 ± 0.87*#	-10%	26.61 ± 1.53	16.64 ± 2.86**####	-37%
HIIE	44.89 ± 0.67	43.29 ± 1.05	-4%	28.64 ± 1.96	23.86 ± 2.73	-17%
SEM+HIIE	42.11 ± 1.51	38.87 ± 1.87 #	-8%	25.18 ± 2.30	12.22 ± 2.14***####	-51%

* indicates comparison with baseline; # indicates comparison with the CON group.
